# Supplementary material for: Ultra-fast charging in aluminum-ion batteries: electric double layers on active anode
Source: Nat Commun. 2021 Feb 5;12:820. doi: 10.1038/s41467-021-21108-4 (PMC7864900; doi:10.1038/s41467-021-21108-4)
Supplement: Supplementary file 1 — Supplementary Information [file 41467_2021_21108_MOESM1_ESM.pdf]

Supplementary Information for

Ultra-fast Charging in Aluminum-Ion Batteries: Electric Double Layers on Active Anode

Xuejing Shen[1,2], Tao Sun[1,2], Lei Yang[1,3], Alexey Krasnoslobodtsev[3,4], Renat Sabirianov[3,4], Michael Sealy[2,4], Wai-Ning Mei[3,4], Zhanjun Wu[1*], and Li Tan[2,4*]

[1]School of Aerospace, Dalian University of Technology, Dalian 116024, China. [2]Department of Mechanical & Materials Engineering, University of Nebraska, Lincoln, NE 68588, USA. [3]Department of Physics, University of Nebraska, Omaha, NE 68182, USA. [4]Nebraska Center for Materials and Nanoscience, University of Nebraska, Lincoln, NE 68588, USA.

These authors contributed equally: Xuejing Shen, Tao Sun.

*Email: Z.W. (wuzhj@dlut.edu.cn); L.T. (ltan4@unl.edu).

Table of Contents

Supplementary Fig. 1. Chemical vapor deposition (CVD) system for graphene growth.........................3

Supplementary Fig. 2. Network of graphene with different textures...4

Supplementary Fig. 3. Anion absorption on graphene...5

Supplementary Fig. 4. Influence from mass and density of 3D graphene to specific capacities.............6

Supplementary Fig. 5. Comparison of pure Al anode and active anode under ultrafast charging........7

Supplementary Fig. 6. Circuit model and data fitting for EIS...8

Supplementary Fig. 7. SEM characterizations of pure Al/Al-LM mesh after full-charging...................9

Supplementary Fig. 8. Liquid metal removed most of the surface defects on Al. 10

Supplementary Fig. 9. The triple Al-complex disrupts the reversible transition between mono- and duo-complex. 11

Supplementary Fig. 10. Cyclic voltammograms (CV) of Ag, Ga, In, Sn, Al and Al-LM.......................12

Supplementary Fig. 11. Cyclic voltammograms (CV) of Ag, Ga, Al and Al-LM without using the 3D graphene cathode...14

Supplementary Fig. 12. Influence of individual metal elements from Galinstan by varying the compositions. 15

Supplementary Fig. 13. Small Ga island covers a small cavity on Al surface. 17

Supplementary Fig. 14. Al(100) and (110) surfaces containing Ga islands....18

Supplementary Fig. 15. The configuration of a "toy" self-diffusion model of ionic liquid covering Al (001) surface in the lowest energy configuration and a bridge position of Al adatom..........................19

Supplementary Fig. 16. The intensity variation with time for $Al_3Cl_{10}^-$ and EMI^+ under current density of 4 A g^{-1}...20

Supplementary Fig. 17. Confocal laser scanning microscopy images of the dendrites growth and dissolution on Al-LM. 21

Supplementary Fig. 18. Raman signals from Al-LM over a wide window of current densities...........22

Supplementary Fig. 19. A more inclusive role for Al triple-complex in discharging. 23
Supplementary Fig. 20. Sampling rate affects reported device properties. 24
Supplementary Note 1. Calculations of theoretical capacity...25
Supplementary References...26

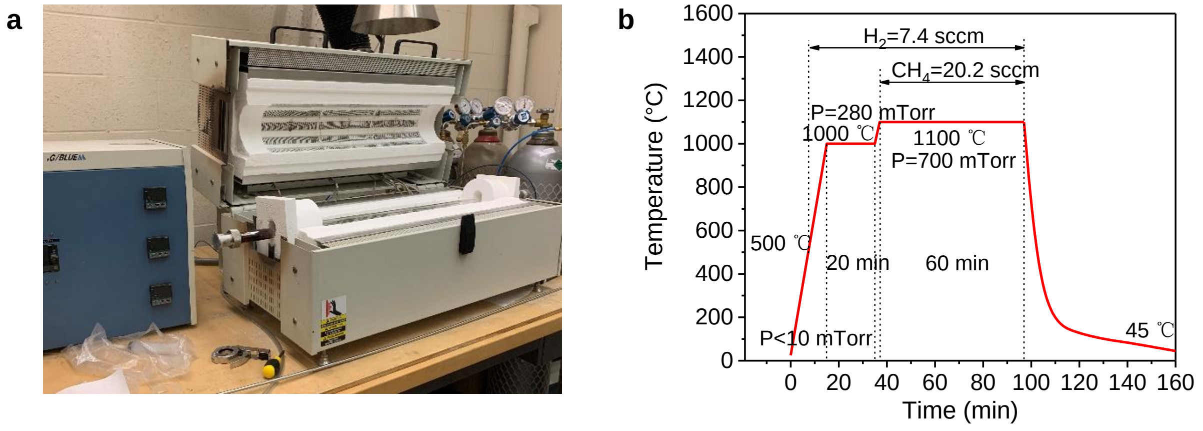

Supplementary Fig. 1. Chemical vapor deposition (CVD) system for graphene growth. The home-built quartz tube furnace (**a**) and control parameters (**b**) used for the growth of graphene.

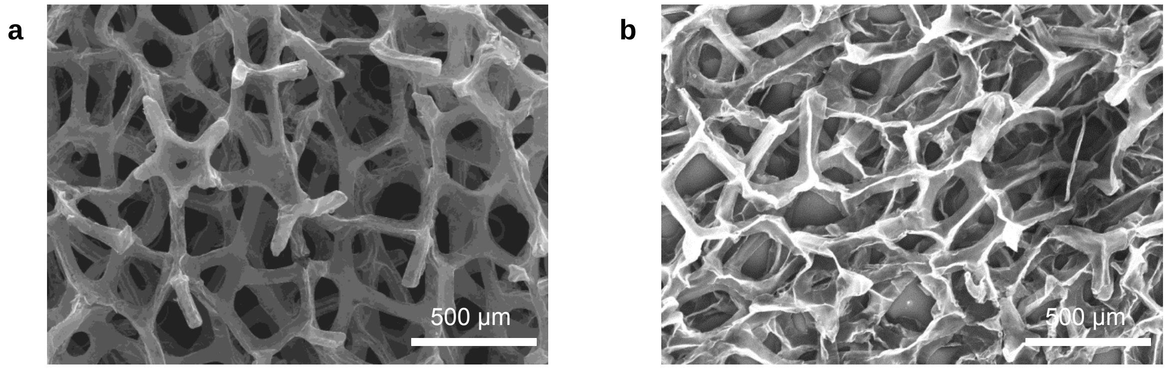

Supplementary Fig. 2. Network of graphene with different textures. SEM images of 3D graphene dried with supercritical CO_2 (**a**) and ethanol (**b**).

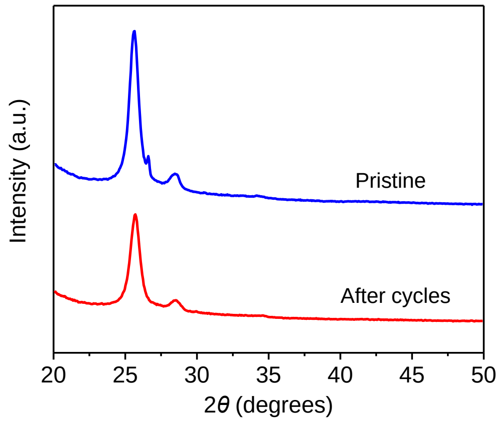

Supplementary Fig. 3. Anion absorption on graphene. Ex situ X-ray diffraction patterns of pristine G-CO_2 and after 1,000 cycles of battery operations. Little change in interlayer spacing suggests anion absorption mainly occurred on open surfaces.

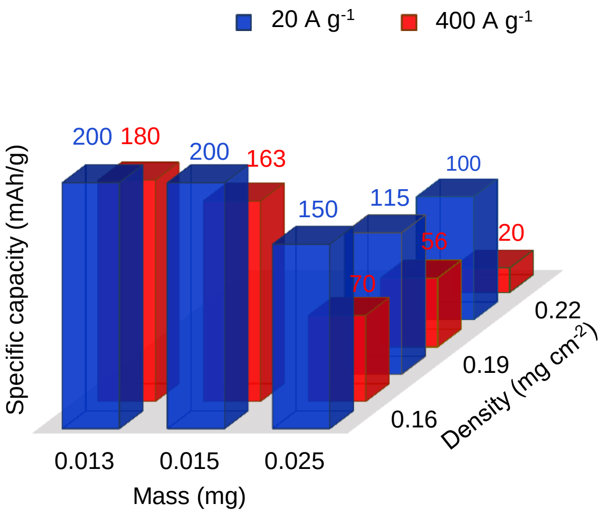

Supplementary Fig. 4. Influence from mass and density of 3D graphene to specific capacities. The specific capacity of a device is affected by two factors: one is the adsorption and desorption of anions from the graphene and the other is the current density on Al anode. The first one becomes more difficult with the increase of carbon density (stacking of graphene layers) and the second one becomes larger as carbon mass increases. The latter will contribute to an elevated surface resistance, making charge transfer less efficient (smaller capacity). In our case, the density is calculated by using the mass of the graphene cathode involved in the reaction divided by the geometric area of this part (rectangular area of the graphene cathode in the top view) that is not the actual surface area of the graphene.

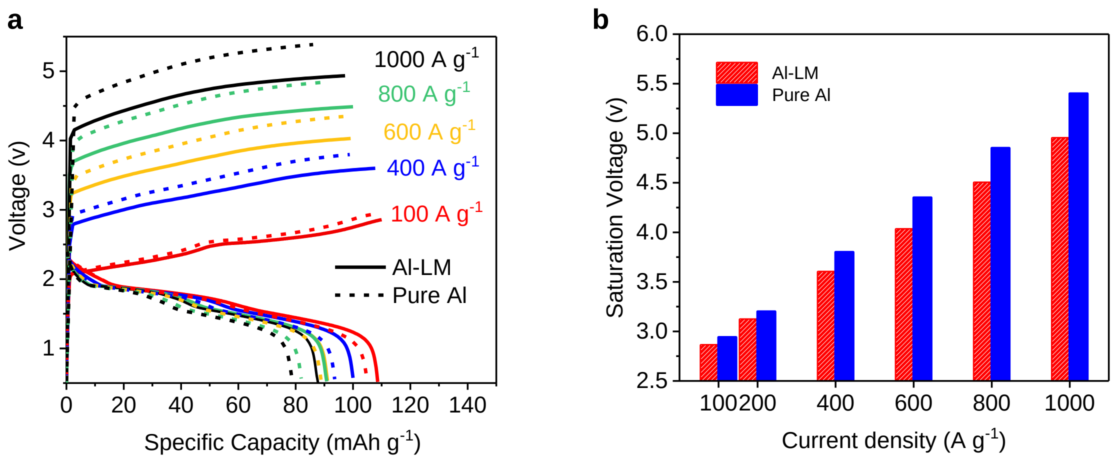

Supplementary Fig. 5. Comparison of pure Al anode and active anode under ultrafast charging. (**a**) Corresponding charge/discharge curves; (**b**) Comparison of saturation voltages using bar graphs of active anode vs. pure Al anode (i_c = 100 ~ 1,000 A g^{-1}, i_{dc} = 100 A g^{-1}). The maximum cut-off voltage with Coulombic efficiency >90% is defined as saturation voltage. Same 3D graphene cathode with the mass of 0.025 mg and density of 0.19 mg cm^{-2} is used in these measurements.

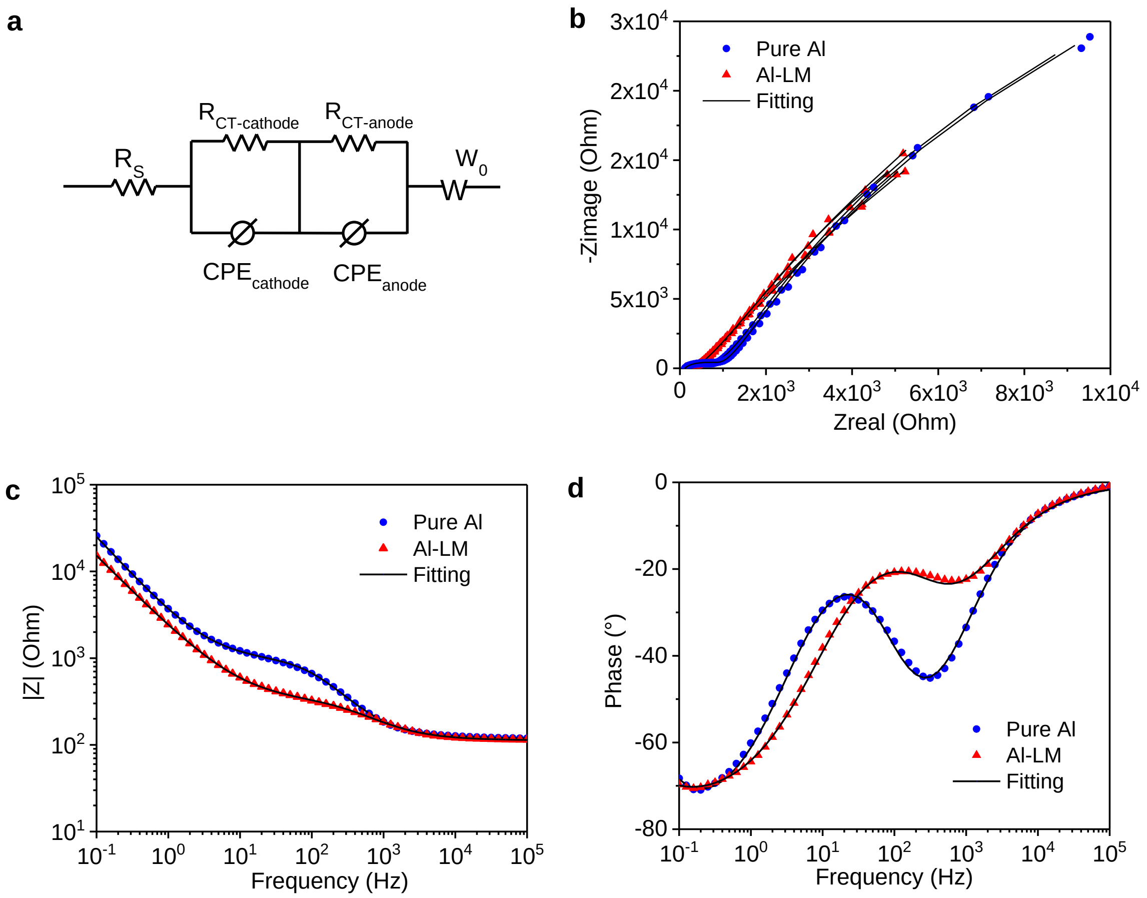

Supplementary Fig. 6. Circuit model and data fitting for EIS. (**a**) Relevant equivalent circuit model for EIS data; (**b**) Nyquist plot; (**c**) Bode plots and (**d**) Bode-phase angle versus frequency plots. The parameter R_S is the electrolyte resistance, constant phase element (CPE) and R_{CT} are the capacitance and charge-transfer resistance, respectively, and W_0 is the Warburg impedance related to the diffusion of ions into the bulk of the electrode. Total of 6 measurements are performed, i.e., four on Al-LM and two on pure Al. All data fitting results are shown in (a) and Figure 2e, and representatives of pure Al anode and active anode were selected respectively to draw their Bode plots (c) and Bode-phase angle versus frequency plots (d), which can show fitting details. Simulated results (solid lines) fitted well with the experimental data (blue and red symbols), indicating the model being reasonable. Resistances for pure Al anode and active anode are calculated with the model, i.e., $R_{CT,\ pure\ Al} = 476.6 \pm 29.60$ ohms; $R_{CT,\ Al\text{-}LM} = 186.5 \pm 17.79$ ohms.

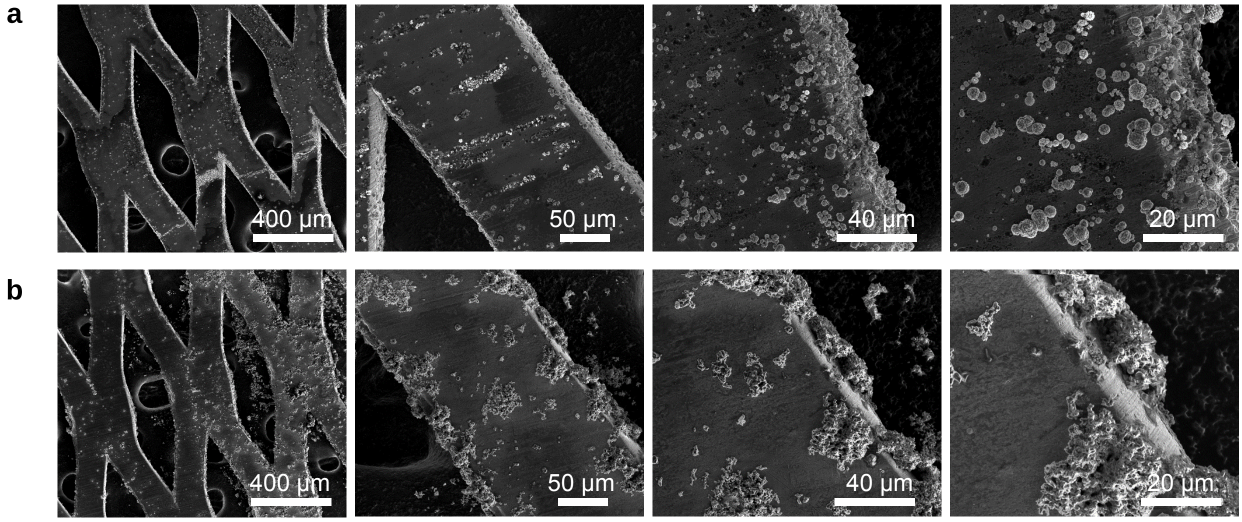

Supplementary Fig. 7. SEM characterizations of pure Al/Al-LM mesh after full-charging. Pure Al mesh (**a**) and Al-LM mesh after 5-min treatment (**b**) under current density of 400 A g^{-1}.

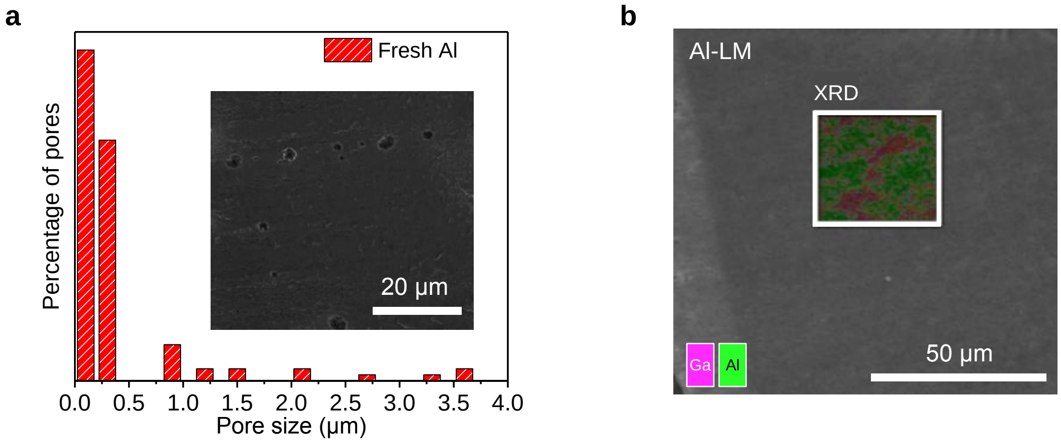

Supplementary Fig. 8. Liquid metal removed most of the surface defects on Al. Quantitative analysis of pores in fresh Al in an area of 60×60 μm^2 (size of the pore in diameter) (**a**), where treated surface barely has anything. SEM images of the fresh Al (**a**, inset) and treated Al (**b**).

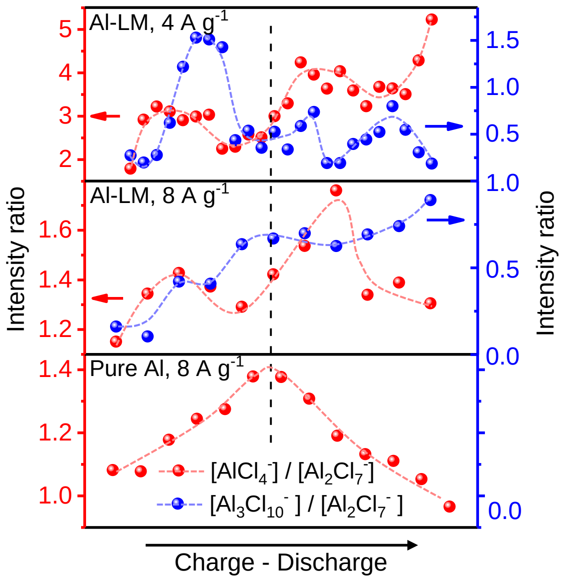

Supplementary Fig. 9. The triple Al-complex disrupts the reversible transition between mono- and duo-complex. Evolution of the ratio between several signature anions that are identified by the Raman spectroscopy.

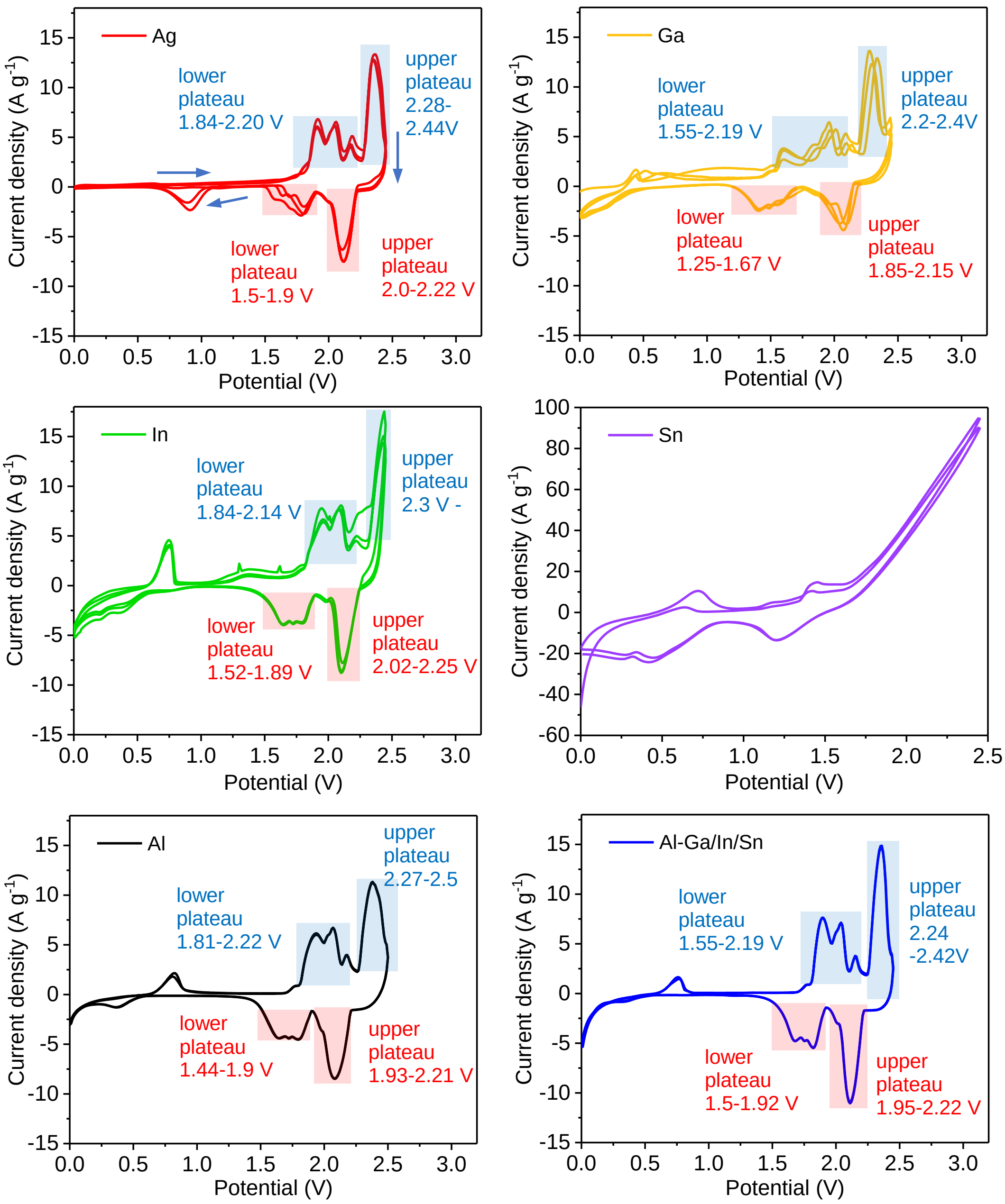

Supplementary Fig. 10. Cyclic voltammograms (CV) of Ag, Ga, In, Sn, Al and Al-LM. The scanning rate is 10 mV s^{-1}. The 3D graphene is the working electrode and Ag/Ga/In/Sn/Al/Al-LM(Ga/In/Sn) as the counter/reference electrode. Major peak around 2.3-2.5 V represents graphene oxidation (accompanied with Al electrodeposition on counter/reference electrode).

Except for tin (Sn) that had an irreversible redox reaction, behaviors of all the other electrochemical cells are rather similar. Here we pay attention to two features: location of the major oxidation peak and repeatability of the entire CV scans (multiple scans performed from 0.0 to 2.5 V). The major peak is the place where $Al^{(0)}$ got electrodeposited on the anode and the 3D graphene cathode was oxidized. Locations for those major peaks varies with different metal anodes, with 2.45 V for In, 2.36 V for Ag, and 2.33 V for Ga. In comparison, Al-LM

showed a complete peak for oxidation at a small potential of 2.35 V. While this number is slightly higher than that for Ga, Al-LM/graphene pair is easier to participate in the redox reactions, with lower oxidation plateaus at 1.55-2.19 V and 2.24-2.42 V but higher reduction plateaus at 1.5-1.92 V and 1.95-2.22 V (see highlights). Essentially, if we translate these plateaus to performance indicators for batteries, devices using Al-LM/graphene will consume the least amount of energy in charging but release the most energy in discharging. Furthermore, a higher current density for the upper plateau (stronger peaks) indicates that the redox reaction is much more intense. In comparison, although the oxidation potential of gallium is the lowest (2.33 V), its reduction platform is also low and peaks are relatively weak in intensity. Next we compare repeatability of the entire CV scans. It represents how well those electrodeposited $Al^{(0)}$ can be oxidized back into the organic electrolyte. As expected, pure Al and Al-LM beat all the other candidates in repeatability, in which little difference is observed for multiple CV scans.

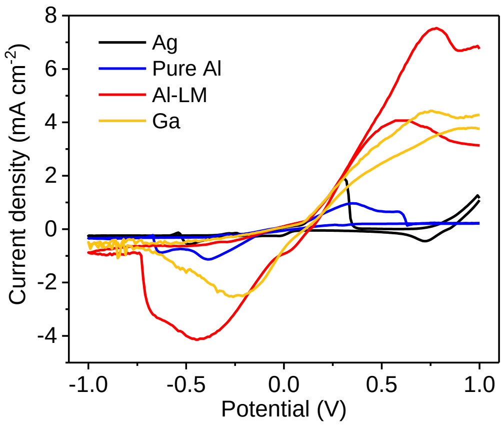

Supplementary Fig. 11. Cyclic voltammograms (CV) of Ag, Ga, Al and Al-LM without using the 3D graphene cathode. The scanning rate is 10 mV s^{-1}. Four different metals were respectively used as the working electrode, in which pure Al was used as the counter/reference electrode. It is clear from these measurements that Al-LM exhibits the highest sensitivity to a given potential (especially comparing to a pure Al electrode), where the reduction process started at the lowest potential among all working electrodes.

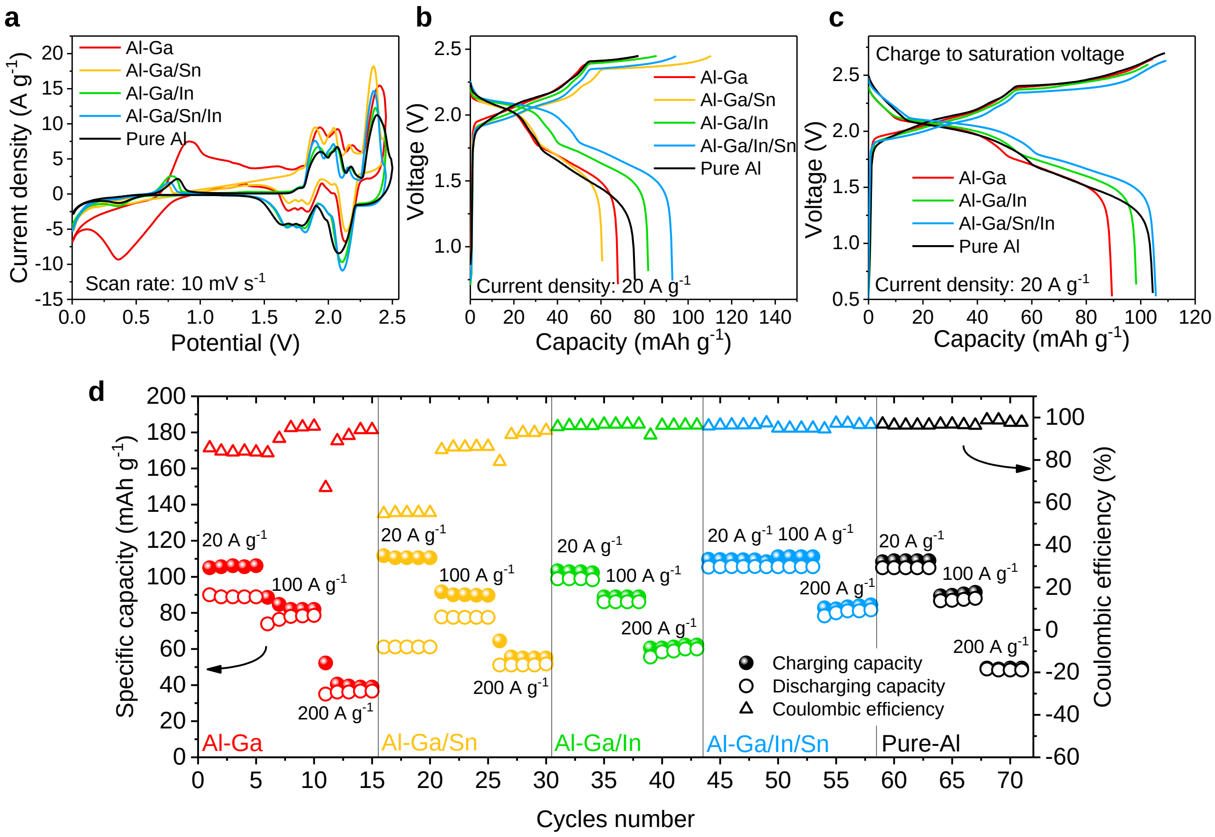

Supplementary Fig. 12. Influence of individual metal elements from Galinstan by varying the compositions. (**a**) Cyclic voltammograms (CV) measured with scanning rate of 10 mV s^{-1}, using 3D graphene as the working electrode and different anodes as the counter/reference electrode. (**b-c**) Galvanostatic charge and discharge curves with different anodes to 2.45 V (b) and their own saturation voltages (c). (**d**) Specific capacities and Coulombic efficiencies of different anodes under saturation voltages. Current densities varied from 20 to 200 A g^{-1}.

We explored the influence of individual metal elements from Galinstan by varying the compositions. Five samples are involved: pure Al, Al treated by pure gallium (Al-Ga), Al treated with eutectic alloy of gallium (75 wt%) and indium (25 wt%) (Al-Ga/Sn), Al treated with eutectic alloy of gallium (85 wt%) and tin (15 wt%) (Al-Ga/In), and Al treated with Galinstan (Al-Ga/Sn/In). Once one of them is used as the anode, we paired it with a 3D-graphene cathode and the organic electrolyte ($AlCl_3$: EMI-Cl = 1.5). Graphs of cyclic voltammogram as well as galvanostatic charge/discharge curves are shown above. Both Al-Ga/Sn and Al-Ga/Sn/In anodes exhibited the lowest value in potential for the major peak at 2.35 V (in (a)), but only Al-Ga/Sn/In had the highest capacity (in (b)) and lowest charging voltage (in (c)). In (d), the battery with Al-Ga/In/Sn demonstrated the best performance in high-rate operations (less decline in capacity). Overall, the liquid metal (Galinstan) we reported in the manuscript is indeed the best anode for Al-ion batteries under high rates.

Now we explain why Al-Ga, Al-Ga/Sn, Al-Ga/In are not as good as Al-Ga/In/Sn. When we performed CV on the single metal (Ga, In, Sn, in Supplementary Fig. 10), we found that gallium (Ga) had the lowest oxidation plateaus (1.55-2.19 V and 2.2-2.4 V), but accompanied

with low reduction plateaus (1.25-1.67 V, 1.85-2.15 V) and weak peaks during discharging. These all suggest that gallium can reduce interfacial resistance, but too much gallium could dissolve freshly deposited Al, discouraging it for subsequent discharging reactions[1]. Additionally, tin (Sn) had signs of irreversible redox reactions, so it plays a negative role in the battery performance. This matches the observation in (d), on anode of Al-Ga/Sn, which has a low Coulombic efficiency. Except for the lack of stability, indium (In) seems to have no obvious drawbacks. However, it exhibited the highest value in potential (higher than our set voltage of 2.5 V; Supplementary Fig. 10). While electrochemically tin is not a favored choice, it does bring down the melting point for liquid metal. Such that, it might have helped a better infiltration through boundaries in aluminum. This is supported by the comparison between Al-Ga/In (Ga : In = 75 : 25 wt%) and Al-Ga/In/Sn in (d).

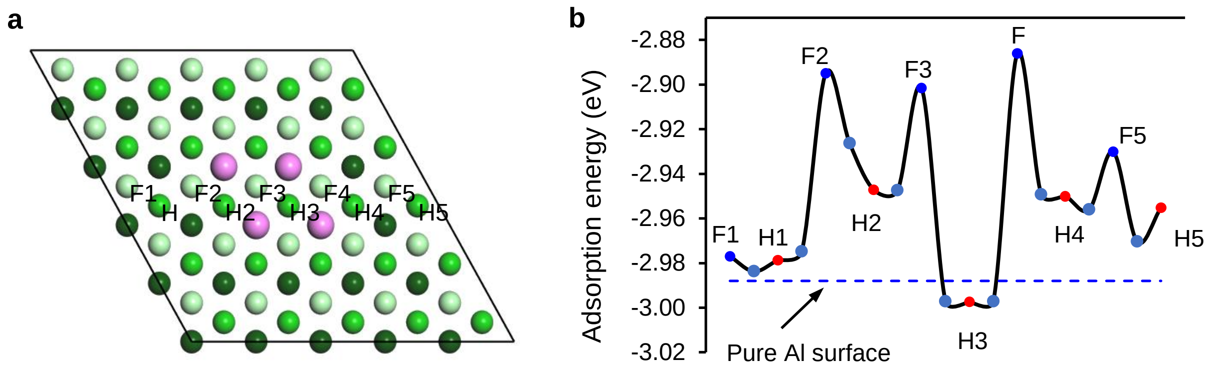

Supplementary Fig. 13. Small Ga island covers a small cavity on Al surface. (**a**) Supercell with a Ga island on top of Al(111) surface; (**b**) the adsorption energy of Al on different hcp, fcc and bridge positions. Al adsorption near the interface of the island can be even lower than the one of Al(111) surface, creating the conditions for potential nucleation.

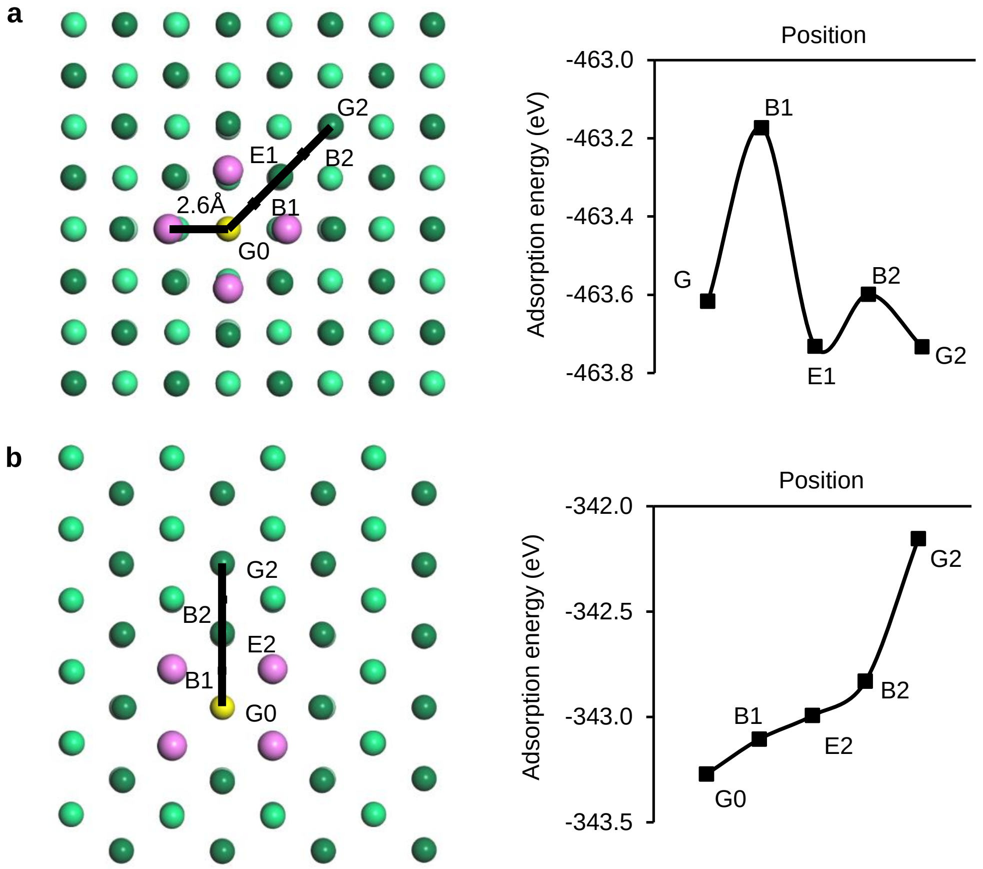

Supplementary Fig. 14. Al(100) and (110) surfaces containing Ga islands. Adsorption energy as function of the position of Al adatom on Al(100) (**a**) and Al(110) (**b**) surfaces in the presence of 4-atom Ga island.

Al(100) and Al(110) are other high symmetry surfaces for Al. The surface energy of these surfaces makes them to be less favorable to occur. However, the diffusion of Al adatom in the presence of Ga island fully supports our conclusions based on consideration of diffusion on (111) surface. Al position at the island reduces its energy comparing to sites on the planar surface of the same symmetry. In case of (110) and (100) surface it is basically due to the bond counting effect as Al creates more bonds when attaches or adsorbs on Ga island. Ga atoms appear to be ready to adjust to optimal Al-adatom (compared to native Al surface that is more rigid in that sense). In case of (111) surface the bond counting considerations does not work by symmetry if Ga island would not deform, however, the direct simulation shows that Ga effectively surrounding Al-adatom providing stronger bonding.

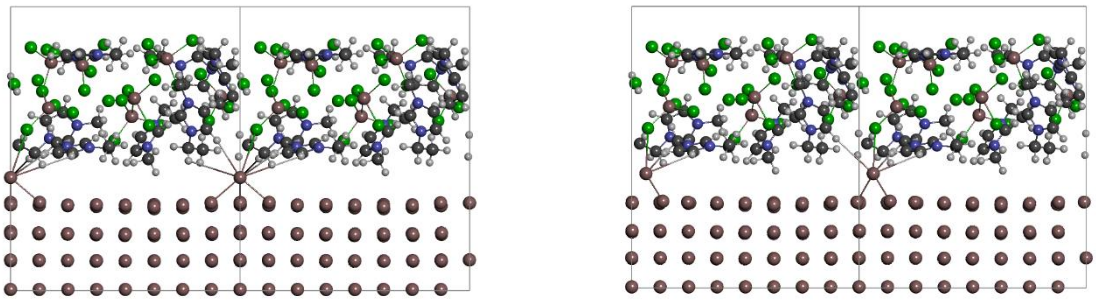

Ground state 4-fold coordinated site

Bridge position 2-fold coordinated site

Supplementary Fig. 15. The configuration of a “toy” self-diffusion model of ionic liquid covering Al (001) surface in the lowest energy configuration and a bridge position of Al adatom. We investigated the effect of ionic liquid on a bridge-hopping diffusion process for (100) surface. Although this is not the lowest energy event, it should be representative of the change in the electrostatic interactions in surface diffusion. (A concerted motion event is expected to be influenced less by IL). We have included seven $EMI^+AlCl_4^-$ complexes at Al (100) surface containing a single adatom using 4×4 supercell with 4 Al layers. We performed a DFT relaxation for the lowest energy position of 4-fold coordinated site. Then we fixed molecular position and considered a bridge-hopping event. By keeping the position fixed we are overestimating the effect of IL on the diffusion process. The results of DFT calculations show that the effect of ionic liquid of the diffusion barrier changes the barrier height from 0.604 to 0.613 eV (in weak electrostatic bonding regime). Although during diffusive events the adatom bonding with the ionic liquid molecules changes, the strength of interaction with ionic liquid is order of magnitude smaller than the interaction of adatom with the surface. The loss of some non-bonding pair interaction during diffusion will be compensated by formation of new non-bonding pair interactions. We are currently investigating approaches to treat the surface electrochemical reactions. There are multiple obstacles of using DFT-based approaches to treat such events. During such processes, the bonding interactions would be introduced and may significantly affect the surface energetics.

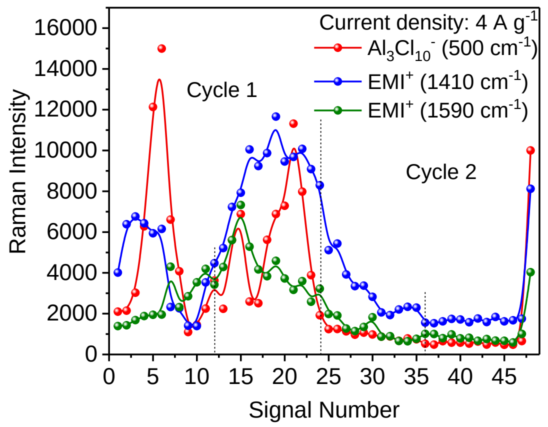

Supplementary Fig. 16. The intensity variation with time for $Al_3Cl_{10}^-$ and EMI^+ under current density of 4 A g^{-1}. A smaller current density here (vs. 8 A g^{-1}) shows a different trend that can be assigned to variability of Raman sensitivity towards surface features on anode (e.g., unevenness and dendrites growth).

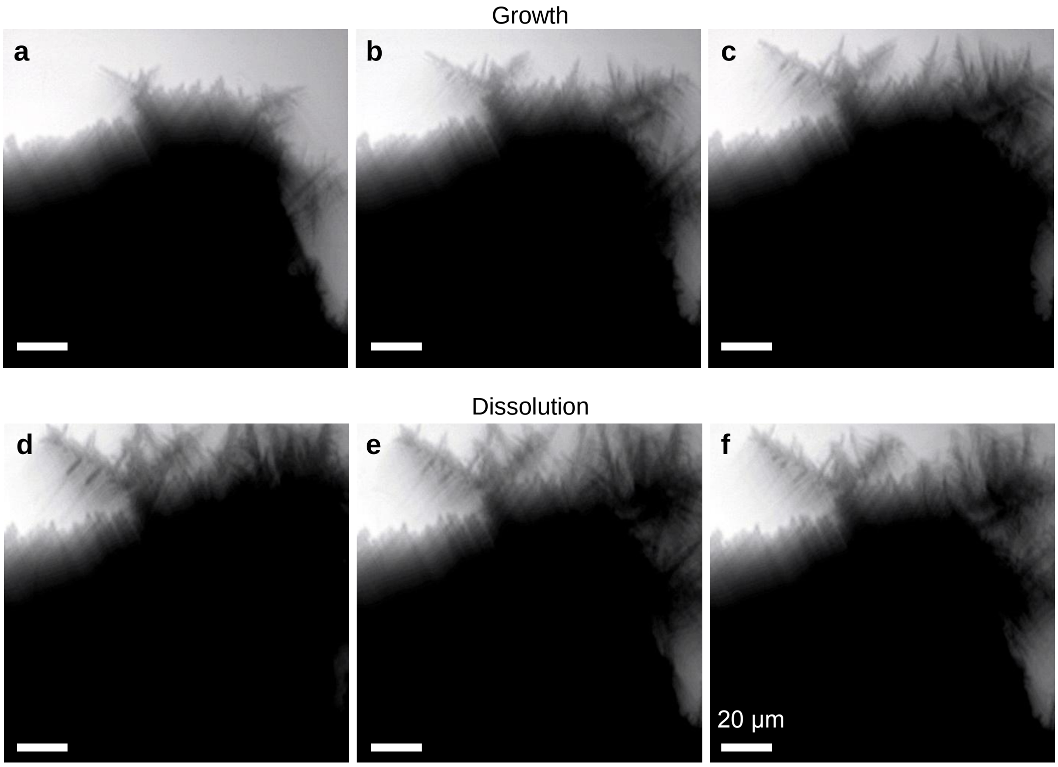

Supplementary Fig. 17. Confocal laser scanning microscopy images of the dendrites growth and dissolution on Al-LM. A planar device with two electrodes, i.e., graphene as the working electrode and Al-LM as the counter/reference, was constructed ($AlCl_3$/EMI-Cl as the electrolyte). A constant current was first applied till a potential of 4.9 V was reached to overcharge this battery (**a-c**) and then it was discharged under the same current (**d-f**). During discharging, the dendrites becomes thinner, resulting in altered curvatures of their branches and trunks. Fine features of the dendrites will contribute to stronger electric field and, hence, larger enhancements factors and as a result an exceptional sensitivity of Raman detection for small amount of molecular/ionic species (EMI^+ and others). Scale bars: 20 μm.

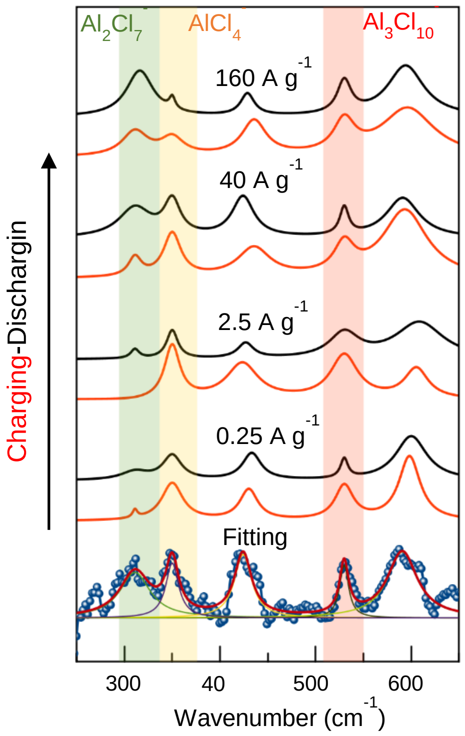

Supplementary Fig. 18. Raman signals from Al-LM over a wide window of current densities. We sampled spectra with a large variation in current density, utilized high power laser with 647 nm excitation, and used pure Al as working electrode (instead of graphene). These modifications allowed for sufficient amount of current (or current density per gram of graphene) to flow through the Al-LM (counter/reference) while still make it possible to capture interpretable Raman signals. These factors as well as large reflection from the Al-LM electrode resulted in small intensities of the signal over the entire spectral range. We have focused our analysis on the 250-650 cm^{-1} window where Al complexes are observed by performing fitting each peak with Lorentzian function for clarity.

High-wavenumber-shift was observed for all the peaks with this experimental setup. The following peaks are assigned: 598 cm^{-1} - EMI^+, 311 and 350 cm^{-1} to $Al_2Cl_7^-$ and $AlCl_4^-$ respectively, and 529 cm^{-1} to $Al_3Cl_{10}^-$. Slightly larger shift for $Al_3Cl_{10}^-$ might indicate further degree of polymerization while staying in the range of peaks between 480 and 540 cm^{-1} typically assigned to $Al_3Cl_{10}^-$.[2] This gallium rich Al-LM (Al-LM_{HIGH}) resulted in the prominent appearance of Al triple-complexes. The peak intensity does follow similar trend as with relatively low gallium content Al-LM (Al-LM_{LOW}; Figure 3 of the manuscript). Although, strengthening and weakening during charging and discharging, this peak never really disappears under the conditions tested for all current densities, which further validates our hypothesis indicating active involvement of the Al triple-complex in electrochemical reactions.

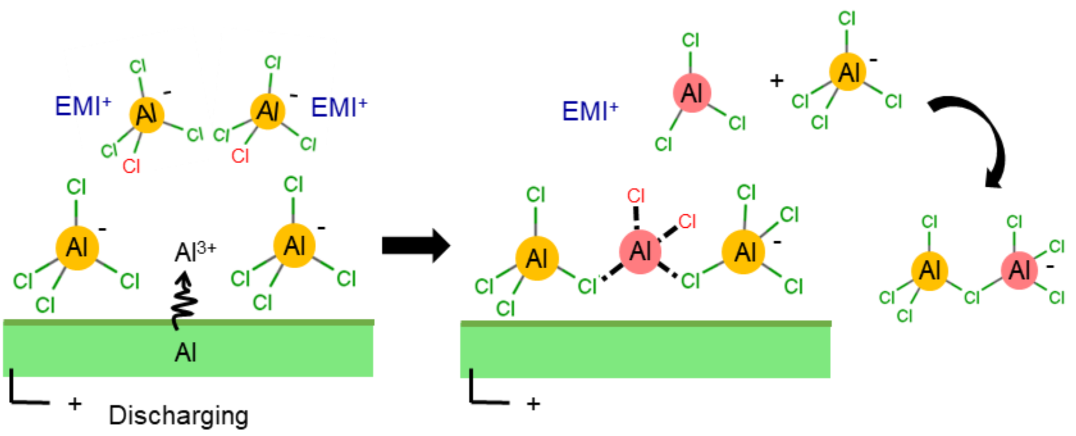

Supplementary Fig. 19. A more inclusive role for Al triple-complex in discharging. This proposed reaction consumes Al^{3+} and Al single-complexes ($AlCl_4^-$) but generates triple-complex ($Al_3Cl_{10}^-$), dual-complex ($Al_2Cl_7^-$), and frees EMI^+ from the bulk electrolyte (EMI^+-$AlCl_4^-$). This entire process is reversed during charging, from right to left.

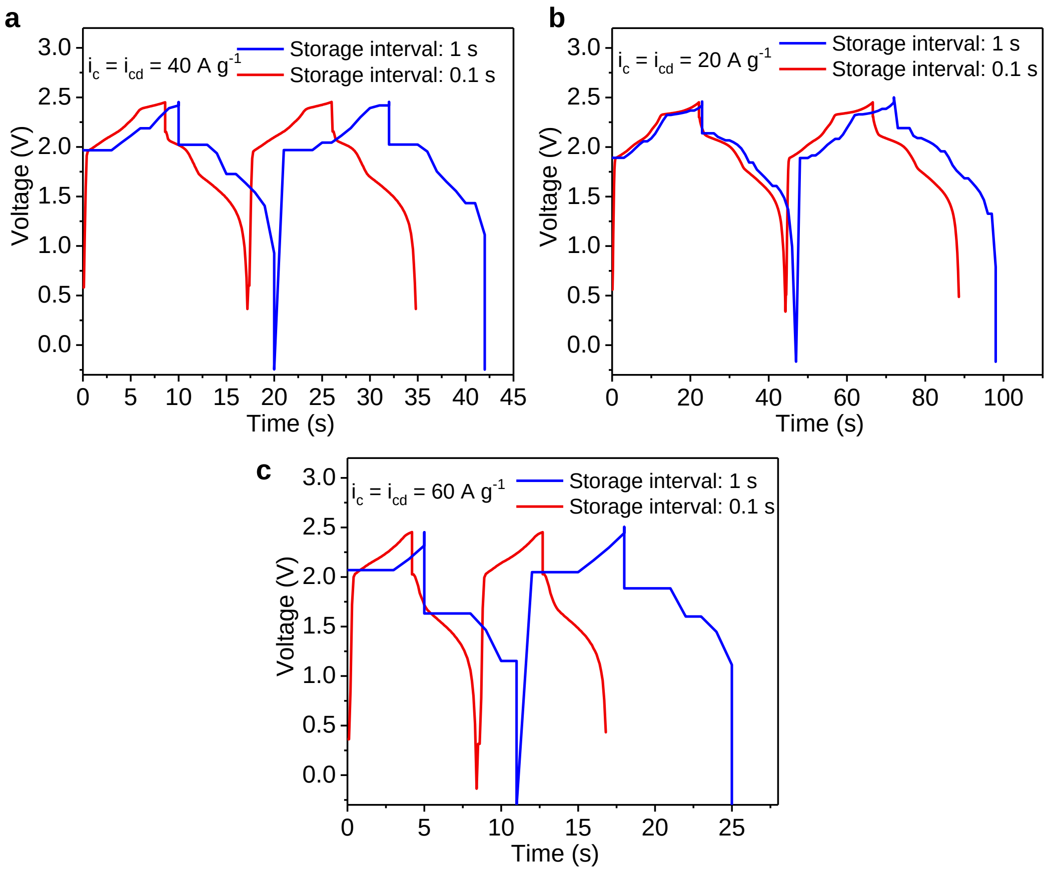

Supplementary Fig. 20. Sampling rate affects reported device properties. Galvanostatic charge and discharge curves under current densities of 20 (**a**), 40 (**b**) and 60 A g^{-1} (**c**) measured by two battery stations: Neware BTS-4008 (50 mA; Minimum data storage interval: 0.1 s) and Neware BTS-3008 (5 mA; Minimum data storage interval: 1 s). While rarely mentioned in literatures, this graph shows sampling rate being a critical factor. Instruments having a small rate would give a rather large number in specific capacity or, an inappropriate sampling rate could mislead the audiences. Essentially for devices running under large current densities, both charging and discharging become quick, demanding a faster sampling rate. In order to provide a fair ground, we used an electrochemical analyzer (CH Instruments, CHI6062E; minimum data interval: 0.1 ms) for all the data received under large current densities.

Supplementary Note 1. Calculations of theoretical capacity

The charge storage capacity is related to the number of ions adsorbed on the cathode. 3D graphene grown on nickel foam has a large surface area but with few stacked layers. From the XRD on the cathode before and after the charging, not much change in interlayer spacing was observed. We therefore conclude most of the absorptions for chloroaluminate ($AlCl_4^-$) occurred on open surfaces of graphene. Let us estimate the capacity using a single layer of anions on one graphene monolayer:

We consider the C-C length in graphene with l = 0.142 nm and the area of a hexagon is:

$$S_{\mathrm{hexagon-G}} = \frac{3\sqrt{3}l^2}{2} = 5.239 \times 10^{-20}\ m^2$$

In each hexagon, there are 2 carbon atoms (1/3*6) so the specific surface area for a single graphene layer (just one side) is:

$$S_{\mathrm{G}} = \frac{S_{\mathrm{hexagon-G}}}{2 * mass\ of\ carbon} = \frac{5.239 \times 10^{-20}\ m^2}{2 \times 1.994 \times 10^{-23}\ g} = 1.314 \times 10^3\ m^2 g^{-1}$$

Next, we take the size of $AlCl_4^-$ as d = 0.479 nm [3] and assume these Al mono-complexes are closely packed on one-side of a monolayer of graphene. We treat them as a center-filled anionic hexagon, where the area is:

$$S_{\mathrm{hexagon-anion}} = \frac{3\sqrt{3}(d)^2}{2} = 5.961 \times 10^{-19}\ m^2$$

In each hexagon, there will be 3 $AlCl_4^-$ complexes (1/3*6 + 1) so the number of close-packed $AlCl_4^-$ per gram of graphene is:

$$N_{\mathrm{anion}} = \frac{3S_G}{S_{\mathrm{hexagon-anion}}} = 6.613 \times 10^{21}\ g^{-1}$$

Theoretical capacity (Q) can be calculated using the Faraday's law, where the number of charge per anion is 1 (for n), F is the Faraday constant, and NA is the Avogadro's constant:

$$Q_{\mathrm{theoretical}} = \frac{nFN_{\mathrm{anion}}}{N_{\mathrm{A}}} = \frac{96485.3329\ sA\ mol^{-1} \times 6.613 \times 10^{21}\ g^{-1}}{6.02214 \times 10^{23}\ mol^{-1}} = 1059.52\ sA\ g^{-1} = 294.31\ mAh\ g^{-1}$$

Considering that the graphene we made has an open 3D network. Graphene layers are not tightly packed, hence most of absorption will happen on the exposed surfaces. Besides, we did not count the edges from graphene in adsorbing anions. Adding all these factors together, specific capacity can be much greater than 294 mAh g^{-1}. Therefore, our specific capacity of 200 mAh g^{-1} is not unreasonable.

Supplementary References

1 Jiao, H. et al. Liquid gallium as long cycle life and recyclable negative electrode for Al-ion batteries. *Chem Eng J* **391**, 123594 (2020).
2 Dymek, C. J. J. et al. ChemInform Abstract: Spectral Identification of $Al_3Cl_{10}^-$ in 1-Methyl-3-ethylimidazolium Chloroaluminate Molten Salt. *ChemInform* **19** (1988).
3 Wang, D. Y. et al. Advanced rechargeable aluminium ion battery with a high-quality natural graphite cathode. *Nat. Commun.* **8,** 14283 (2017).
